# Revisiting Kadenbach: Electron flux rate through cytochrome c‐oxidase determines the ATP‐inhibitory effect and subsequent production of ROS

**DOI:** 10.1002/bies.201600043

**Published:** 2016-05-12

**Authors:** Sebastian Vogt, Annika Rhiel, Petra Weber, Rabia Ramzan

**Affiliations:** ^1^ Cardiovascular Research Lab, Biochemical Pharmacological Research CenterPhilipps‐University MarburgMarburgGermany

**Keywords:** allosteric inhibition, cytochrome c oxidase, enzyme kinetics, ischaemic preconditioning, phosphodiesterase inhibitors, reactive oxygen species

## Abstract

Mitochondrial respiration is the predominant source of ATP. Excessive rates of electron transport cause a higher production of harmful reactive oxygen species (ROS). There are two regulatory mechanisms known. The first, according to *Mitchel*, is dependent on the mitochondrial membrane potential that drives ATP synthase for ATP production, and the second, the *Kadenbach* mechanism, is focussed on the binding of ATP to Cytochrome c Oxidase (CytOx) at high ATP/ADP ratios, which results in an allosteric conformational change to CytOx, causing inhibition. In times of stress, ATP‐dependent inhibition is switched off and the activity of CytOx is exclusively determined by the membrane potential, leading to an increase in ROS production. The second mechanism for respiratory control depends on the quantity of electron transfer to the Heme aa3 of CytOx. When ATP is bound to CytOx the enzyme is inhibited, and ROS formation is decreased, although the mitochondrial membrane potential is increased.

**Abbreviations**
CytOx: cytochrome c oxidase (E.C. 1.9.3.1.)
H: Hill slope coefficient (according to GraphPad Prism software)
PEP: phosphoenolpyruvic acid
PK: pyruvate kinase (E.C. 1.7.1.40)
ROS: reactive oxygen species
TN: turn over number

## Introduction

Mitochondrial respiration is the most common mechanism for ATP production and energy supply. Energy demand varies and depends on different forms of activities in cells, tissues and organisms. Early in earth's history, there were Prokaryotes that were dependent on their own metabolism and on nutrients that were provided by their environment. Approximately 250 million years ago, after dramatic changes occurred in earth's atmosphere, its composition changed to 21% oxygen 1. The altered conditions led to a change in the production of cellular energy from anaerobic glycolysis to respiration because a huge energy supply was required for the survival of Eukaryotes 2. The question of adequate regulation of respiration appeared. As is known today, regulation of respiration is absolutely essential to minimise potentially harmful by‐products, including reactive oxygen species (ROS). The factors that adapt respiration to physiological strain remain poorly understood (Box 1).

Box 1 **Mitochondria are the ‘power houses’ of the cell**

Cellular respiration involves oxygen consumption to produce ATP. The mitochondrial electron transport chain (ETC) is composed of five elements: Four enzymatic respiratory complexes and ATP synthase. Electrons that are delivered from NADH and succinate pass through the electron transport chain to oxygen, which is reduced to water. Electron transmission between respiratory chain complexes liberates free enthalpies (Δ*G*°’) of −50 kJ/mol (NADH to the Fe‐S centre of NADH‐Q Reductase), −42 kJ/mol (Cytochrome b to Cyt c1 in Cytochrome – Reductase) and −100 kJ/mol (Cytochrome a to oxygen in CytOx) 3. Every single step of electron transmission releases enough free enthalpy to drive ATP synthase (−30.5 kJ/mol). The reactions of the substances involved require catalysis and the control of energy, which is then released in a stepwise manner. Electrons that are released from aerobic glycolysis and the Krebs’ cycle are transferred to the mitochondrial respiratory chain located on the inner mitochondrial membrane. Within the mitochondrial intermembrane space, hydrogen ions accumulate by the action of proton pumps. The mitochondrial enzyme complexes I, III and IV are proton pumps that are involved in maintaining a mitochondrial membrane potential (ΔΨm), which supplies energy for the rotation of the ATP synthase (complex V), resulting in ATP production. Energy supply is related to the energy demand of tissues. However, the issue regarding the limitation of the increase of the membrane potential as it relates to the accelerated production of ATP and increased ROS production has to be addressed, although a mitochondrial transhydrogenase normally regenerates NADPH from NADH to increase the antioxidative capacity. In pathological myocardial workload conditions this reaction was observed to be reversed, resulting in NADPH depletion with increased production of ROS 4.

Regulatory mechanisms that coordinate energy production are required. The current theory says there are two steps for the regulation of production of high energy phosphate compounds. First, proton pumping by mitochondrial enzyme complexes I, III and IV results in the establishment of a mitochondrial membrane potential (ΔΨm). This potential drives the ATP synthase (complex V) toward production of ATP (Mitchell‐theory). In a second feedback mechanism, the binding of ATP to mitochondrial complex IV results in an allosteric inhibition of Cytochrome c Oxidase (CytOx) activity (Kadenbach theory), thus inhibiting excessive rates of ROS production, which could render the mechanism a pivotal element understanding degenerative diseases, and in turn, for developing modern therapeutic concepts 5. Since 1997 the second mechanism of respiratory control (see Box 2) has remained controversial because the measurements were questionable.

Box 2 **Current view of Kadenbach's theory as an extension of Mitchell's theory**

The molecular mechanisms are currently not fully understood. With regard to the present concepts see Fig. 2A–D.

1. As is generally known, electrons are transferred through the ETC (from complex I to IV). Additionally, complexes I, III and IV act as proton pumps, translocating hydrogen ions (protons) across the inner mitochondrial membrane and creating a mitochondrial membrane potential. This ΔΨm drives complex V (ATP‐synthase) toward production of ATP. As reported by Kaim and Dimroth, an optimal range between 80 mV <ΔΨm <120 mV guarantees sufficient synthesis of ATP, resulting in the transfer of 4 electrons to dioxygen (reduction) and oxidation of hydrogen to water without excessive production of ROS. At ΔΨm >>120 mV, large amounts of ROS are produced 5, 63. In our measurements using isolated myocardial mitochondria, we have found greater increased values of ΔΨm, thus we assume a permanent release of ROS under such experimental settings in vitro.
2. Under physiological conditions, the cell contains extremely high quantities of ATP. In the so called ‘relaxed’ state, ATP binds to subunit IV of the phosphorylated CytOx and induces an allosteric conformational change that results in sigmoidal enzyme kinetics. Therefore, CytOx is ‘ATP‐dependent allosterically inhibited’ and an increased production of ROS during respiration is avoided. Although the membrane potential is sustained by the ATP demand, in this state (within a stable range of ΔΨm), the enzymatic activity of CytOx after ATP binding is regulated exclusively by the mitochondrial ATP/ADP ratio.
3. In the case of cellular stress, allosteric inhibition of the enzyme by ATP is switched off. Although the maximum rate of ATP synthase is known to be beyond 100–120 mV 63, this ΔΨm increases because of the higher CytOx activity. Because CytOx is the rate‐limiting step in the ETC, its activity increases for higher ATP production 64. Electron transmission from complexes I to IV is accelerated at closer distances between the complexes because of mitochondrial membrane shifts 65, resulting in maximum production of water with CytOx. Both prokaryotic and eukaryotic ATP synthase complexes have the same capacity with respect to their maximum rates 71. A turnover number for ATP synthesis of 270 ± 40/s was determined in the presence of 5% active F0F1 complexes. In the case of hyperpolarization of ΔΨm, the potential drives ATP synthase beyond its capacity, resulting in the maximum production of ATP, but exceeds effective ‘oxygen utilization’. The ΔΨm exceeds the normal range, and ROS are formed in high concentrations because of the maximum synthesis rate of ATP synthase 63. At this stage, CytOx activity is determined by the ΔΨm, and no longer by the ATP/ADP ratio. In parallel, the concentration of ROS increases, resulting in harmful effects to the cell.
4. Kadenbach's Hypothesis states that the regulation of the membrane potential and ROS formation in mitochondria are determined by the ATP‐induced allosteric inhibition of CytOx, and represents a second mechanism for respiratory control. Under relaxed conditions, feedback inhibition of CytOx by ATP maintains the membrane potential at low values. Stress factors increase the cytosolic and/or mitochondrial [Ca2+], which activates calcium‐dependent protein phosphatases and dephosphorylates CytOx. Without allosteric inhibition of CytOx by ATP, the membrane potential increases with a consequent increase of ROS formation. Question regarding initial mechanisms have yet to be answered. There is a competition between ADP and ATP for the binding sites on the enzyme 66. With reconstituted enzyme, the kinetics were influenced by extraliposomal (cytosolic) ATP and ADP. The Km for cytochrome c was five times higher in the presence of extraliposomal ATP than with ADP. These differences of Km values were abolished after preincubation of the enzyme with a monoclonal antibody to subunit IV. The data demonstrate the regulation of cytochrome c oxidase activity by the cytosolic ATP/ADP ratio, in addition to regulation by the matrix ATP/ADP ratio. Cyclic AMP activation of mitochondrial PKA, which is generated by the carbon dioxide/bicarbonate‐regulated soluble adenylyl cyclase 67 is found to induce phosphorylation of CytOx 68 and to influence the enzymatic activity. Finally, the redox‐dependent transfer of protons to the binuclear centre through the D‐channel and the K‐channel, where the latter is redox‐independent 69 remains to be clarified. Interacting effects of ΔH+ and ΔΨm on ΔpH, as a controlling step in the ETC activity have already been discussed 70. Nevertheless, our data suggest that there is no allosteric inhibition of CytOx when electron transfer is increased. Permanent stress and elevated ROS levels can result in cell apoptosis and the generation of multiple degenerative diseases.

### Polarographic assay of CytOx activity enables detection of an ATP‐dependent inhibitory effect

Respiratory steady states have already been defined by Chance and Williams 6 according to a protocol for oxygraphic experiments with isolated mitochondria corresponding to the activities of all 5 of the mitochondrial multienzyme complexes of the ETC. Ferguson‐Miller et al. 7 reported the polarographic measuring procedure in a measuring cell, which operates according to the principle of the Clark electrode, which is based on *selective* electron transfer by an electron donor (ascorbic acid 18 mM) and an electron transmitter (cytochrome c in increasing concentrations) to the mitochondrial respiratory chain complex IV (CytOx). Subsequently, Kadenbach and co‐workers used this system to perform measurements either in the presence of 5 mM ADP or 5 mM ATP. For measurements in the presence of ATP, an ATP regenerating system (10 mM Phosphoenolpyruvate, 2 U/mL pyruvate kinase, 5 mM MgSO_4_) was also used to maintain the ATP concentrations high enough and to demonstrate the effect of inhibited CytOx.

Studies by Arnold and Kadenbach 8 described the influence of intramitochondrial ATP/ADP ratios with increasing amounts of cytochrome c in the liposomally reconstituted enzyme. An increased ATP to ADP ratio resulted clearly in sigmoidal enzyme kinetic curves (*H*_K_ from 1.09 to 1.97) at increasing cytochrome c concentrations (from 0.25 to 60 µM). However, at high concentrations of cytochrome c, the enzyme kinetics became hyperbolic. In these experiments, CytOx kinetics was measured as ‘Turn over number’ (TN [S^−1^]) 9, 10. CytOx was reconstituted in proteoliposomes at a concentration of 50 nM with varying ratios of ATP/ADP inside the vesicles. Therefore, the aa_3_ content inside the proteoliposomes was constant, but addition of increasing amounts of cytochrome c resulted in a steady increase of electron transmissions to aa_3_ and in higher TN's at the end. According to our hypothesis, the shift from sigmoidal to hyperbolic kinetics described in this report is attributed exclusively to the electron transmission rate to CytOx, whereas the enzymatic consumption of oxygen itself depends on the uptake of electrons by aa3 when increasing amounts of cytochrome c are transferred to dioxygen for reduction 9, 10.

Acceptance of this type of CytOx activity measurements has always been controversial. First, criticisms were based on the fact that original measurements from Arnold and Kadenbach 8, which were performed using a reconstituted enzyme, were not directly comparable to measurements with the isolated enzyme, or measurements with mitochondria or tissues. Second, the common use of N,N,N′,N′‐tetramethyl‐p‐phenylenediamine (TMPD) in enzymatic kinetic measurements hides the electron transfer rate dependency because electrons are transferred not only to cytochrome c but also directly to the enzyme 11 and even bypass the cytochrome c binding site 12. Third, the use of detergents, which is thought to have intermediary effects on enzymatic kinetic measurements for permeation of cytochrome c through the outer mitochondrial membrane, is rather deleterious. Detergents destroy the mitochondrial membrane's architecture and ‘create’ a mixture of isolated enzymes, mitochondrial and tissue fragments as well. Different forms of agglutinates were observed by electron microscopy. The activity of membrane bound enzymes varies in a wide range. The activity of the purified enzyme is partially inhibited by Triton X‐100 and dramatically enhanced by Tween 80 or phospholipids 13.

**Figure 1:**
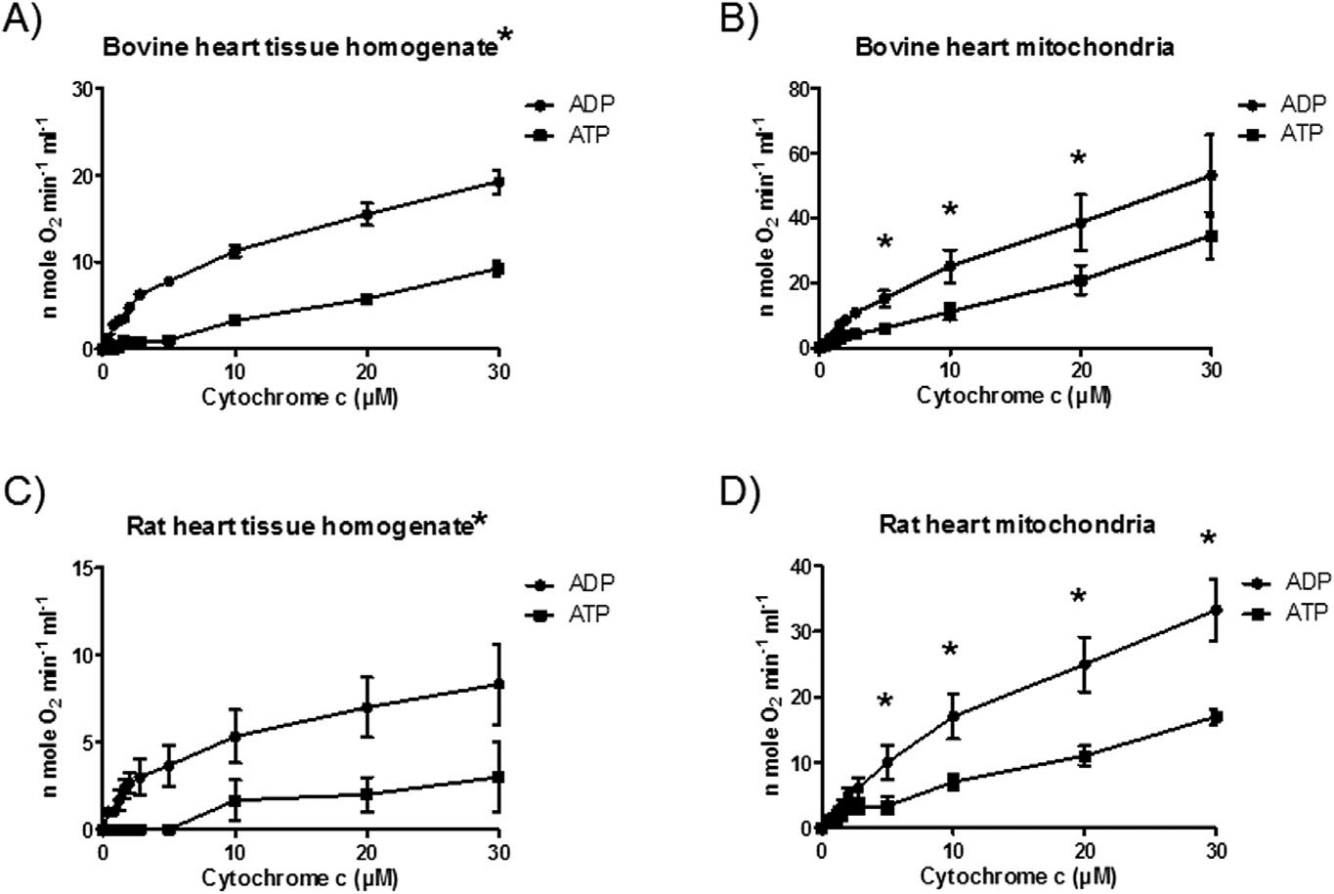
Polarographic measurements of CytOx kinetics in bovine heart tissue homogenate (**A**) and mitochondria (**B**). Bovine heart tissue that was frozen at −80 °C was thawed, homogenized on ice in 5 volumes of standard isolation buffer (250 mM sucrose, 20 mM Hepes, 1 mM EDTA, pH 7.4) and used directly for kinetic measurements of CytOx activity or subjected to isolation of mitochondria by standard isolation procedures 14. Rats were sacrificed by decapitation and rat hearts were homogenized in the standard isolation medium (as used in the bovine heart procedure, but in addition contained 0.2% fatty acid‐free BSA) using a homogenizer after cutting the tissue into small pieces using a scissor. This rat heart tissue homogenate was either used directly for kinetic measurements of CytOx activity (**C**) or used for the isolation of mitochondria (**D**). Kinetic measurements were performed in the sucrose buffer in the presence of 5 mM ADP or 5 mM ATP, 10 mM Phosphoenolpyruvate and 160 U/mL pyruvate kinase at increasing concentrations of cytochrome c, along with 18 mM ascorbate to reduce cytochrome c. The concentration of aa_3_ in the bovine heart tissue homogenate that was used for oxygen measurements was 113.57 ± 7.73 nM, and that used in the bovine heart mitochondria was 567.84 ± 38.76 nM and was determined spectrophotometrically (*n* =4). Although the aa3 content in the rat heart tissue homogenate was 48 ± 5.54 nM, in the rat heart mitochondria it was 251.32 ± 20.16 (*n* = 3). *Wilcoxon‐Mann‐Whitney‐rank sum test *p* < 0.05.

The latter two factors especially were considered for a long time as interfering factors for efficient kinetic measurements of the enzyme. Effective measurements are achieved only under very stringent conditions. We performed very simple experiments (supplemental data in 14) using bovine heart tissue homogenate and isolated bovine heart mitochondria (Fig. 1A and B), as well as rat heart tissue homogenate and isolated rat heart mitochondria (Fig. 1A–D). In all cases, measurement conditions were standardised as mentioned previously 14, and reproducible results were achieved. Interestingly, measurements using homogenates in both cases consistently demonstrated significant allosteric ATP‐dependent enzyme inhibition, in contrast to results with isolated mitochondria. The term ‘ATP‐dependent enzyme inhibition’ is primarily observed at low, rather physiological intramembrane concentrations of Cytochrome c 15, 16, and subsequent to the performed kinetic experiments. In these experimental settings, spectrophotometric analysis revealed a difference in aa_3_ content at the same protein ratio of 1–2.25 between homogenate and mitochondria. During measurements of CytOx kinetics, we employed experimental conditions that were similar to those reported by Arnold and Kadenbach 8. Different rates of electron transfer from increased cytochrome c concentrations to the aa_3_ inside the CytOx molecule created a pivotal point for understanding the inhibitory effect of ATP. Hill‐slope (H) values were calculated using an allosteric sigmoidal programme (GraphPad Prism software) as a parameter of allostery, where *H* = 1 indicates that the equation is identical to standard Michalis‐Menton kinetics. Hyperbolic curves were obtained in all ADP‐measurements. When H is greater than 1, the curve is sigmoidal due to positive cooperativity, as in the presence of ATP and an ATP‐regenerating system. We observed sigmoidal curves during tissue homogenate measurements of both rat heart and bovine heart. (Table 1, Fig. 1A–D). Consequently, polarographic measurements performed with different concentrations of freshly isolated mitochondria showed altered enzyme kinetics with dilution series of mitochondria (Ramzan et al., unpublished results). Variations of involved components (ascorbate, cytochrome c) confirmed these results. Therefore, we conclude that the ‘Kadenbach effect’ is triggered by two main components: (i) the extremely high amount of ATP that is present in intracellular high‐energy phosphates, and (ii) the number of electrons transmitted from cytochrome c to aa3 of CytOx.

**Table 1:** Using an allosteric sigmoidal programme in GraphPad Prism software, Hill‐slope (H) calculations were performed using the CytOx kinetics shown in Fig. 1A–D

Samples	ADP (H)	ATP (H)
Bovine heart tissue homogenate (*n* = 4)	0.8899	∼1.103
Bovine heart mitochondria (*n* = 4)	0.9802	0.8115
Rat heart tissue homogenate (*n* = 3)	0.5566	1.152
Rat heart mitochondria (*n* = 3)	0.8868	0.8062

When H > 1 as shown in Table 1, the kinetics of the enzyme are allosteric as is apparent in Fig. 1A–D. When H < 1, the curve is hyperbolic in accord with the Michaelis Mention equation.

Finally, the effect of phosphorylation and the importance of individual cytosolic signal pathways switching this effect on or off are not yet fully understood. In our experiments, we failed to reproduce the initial data from Lee, where allosteric inhibition of CytOx was induced by Protein kinase A and switched off by Phosphatase PP1 9, 10. Various factors, for example the presence of phosphatases/phosphatase inhibitors or protein kinases/protein kinase inhibitors were used in the polarographic measurements to initiate or modify allosteric inhibition of CytOx, which however, yielded no clear results. Regulatory roles of specific phosphorylation sites of mitochondrial proteins are often neither discussed nor analysed in detail, especially in reports of ischaemic myocardial pathophysiology 17, although mitochondrial protein kinases and phosphatases and their substrates participate 18, 19 in apoptosis and in the formation of infarct tissue 20, 21, 22. A search for the molecular cause of cardiac injury after ischaemia must focus on mitochondria 18, 23, 24 and their production of ROS.

Kadenbach's theory provides an explanation for the regulation of ROS production in mitochondria. ROS are considered the main cause of development of degenerative diseases 25, for damaging the heart after ischaemia 26, and in development and progression of heart failure (Box 3) 27, 28, 29, 30, 31, 32.

The production of ROS is dependent on the regulation of the mitochondrial membrane potential (ΔΨm). The theory of Kadenbach describes a mechanism that maintains the ΔΨm at low values under normal conditions, thus preventing excessive formation of ROS. This mechanism is switched off under conditions of stress and excessive work to maximise the rate of ATP synthesis and is accompanied by a decreased efficiency 56. There is a physiological balance between oxidative and reductive processes in biological systems, and the constant relationship between both is maintained by an ‘antioxidative capacity (AOC)’. A predominance of reactive oxygen intermediates is referred to as ‘oxidative stress’. ROS are scavenged by corresponding enzyme systems that are classified as either enzymatic or non‐enzymatic 57, 58. This classification has clinical value because the regulation of redox reactions is important for protecting the heart from coronary disease 59, 60. These observations conform to the work of Prosser et al. 61 who found that production of ROS is induced when cardiac cells are physiologically strained. Additionally, the induction of protein neogenesis and protein assembly of mitochondrial proteins has also been assumed (Box 3) 62.

Box 3 **ROS have double functions**

Small amounts act as ‘secondary messengers’ and are probably formed by cells after binding of agonists (e.g. EGF, interleukins, TNF‐α) to receptors of signaling cascades through NADPH oxidases at the plasma membrane 33. They act on receptor tyrosine kinases, protein kinase C, or on mitogen‐activated protein kinases, such as those involved in signaling pathways of ERK1/2, JNK, or directly on transcription factors, in addition to leading to changes in calcium homeostasis, cellular pH or the degree of reduction of nucleotides (NADH/NAD+) and ATP content 23, 34, 35, 36. Otherwise, they appear in mitochondria at high membrane potential (Ψm >140 mV) values 37, 38. ROS can also trigger apoptosis by the release of cytochrome c through lipid peroxidation or by opening of the mitochondrial permeability transition pore (MPTP) 39, 40, 41. They can also chemically modify proteins, lipids and nucleic acids. The dual role of ROS in the regulation of life and death (apoptosis, necrosis) of cardiomyocytes during ischaemia/reoxygenation was discussed by Das 42.

Measurements of ROS formation at high Ψm values were performed in isolated mitochondria and were additionally confirmed in cultured cells 43, 44, 45. Normally, in isolated mitochondria and in reconstituted cytochrome c oxidase 46, which is the terminal enzyme of the mitochondrial respiratory chain, high values (140–200 mV) of Ψm are observed. However, low Ψm values are found in perfused rat hearts (100–140 mV) 47, 48, cultured fibroblasts (105 mV) and neuroblastoma cells (81 mV) 49. Kadenbach's theory explained this discrepancy in a new hypothesis for the regulation of mitochondrial energy metabolism in living cells 9, 10, 50, 51, 52, 53. This hypothesis explains the increase of Ψm and the formation of ROS in mitochondria under stress conditions and excessive work 54. On this issue, the influence of an intramembrane ΔpH (ΔH+) shift on the proton motion force (ΔP) remains an open question. Kim et al. determined the contribution of ΔΨm and ΔH+ to ΔP split to 153:66 mV in state 4, and 137:38 mV in state 3. However, valinomycin tends to redistribute the components of ΔP to favour a higher ΔH+ and a lower ΔΨm, even in low potassium media 55.

### Signaling factors and CytOx interactions

**Figure 2:**
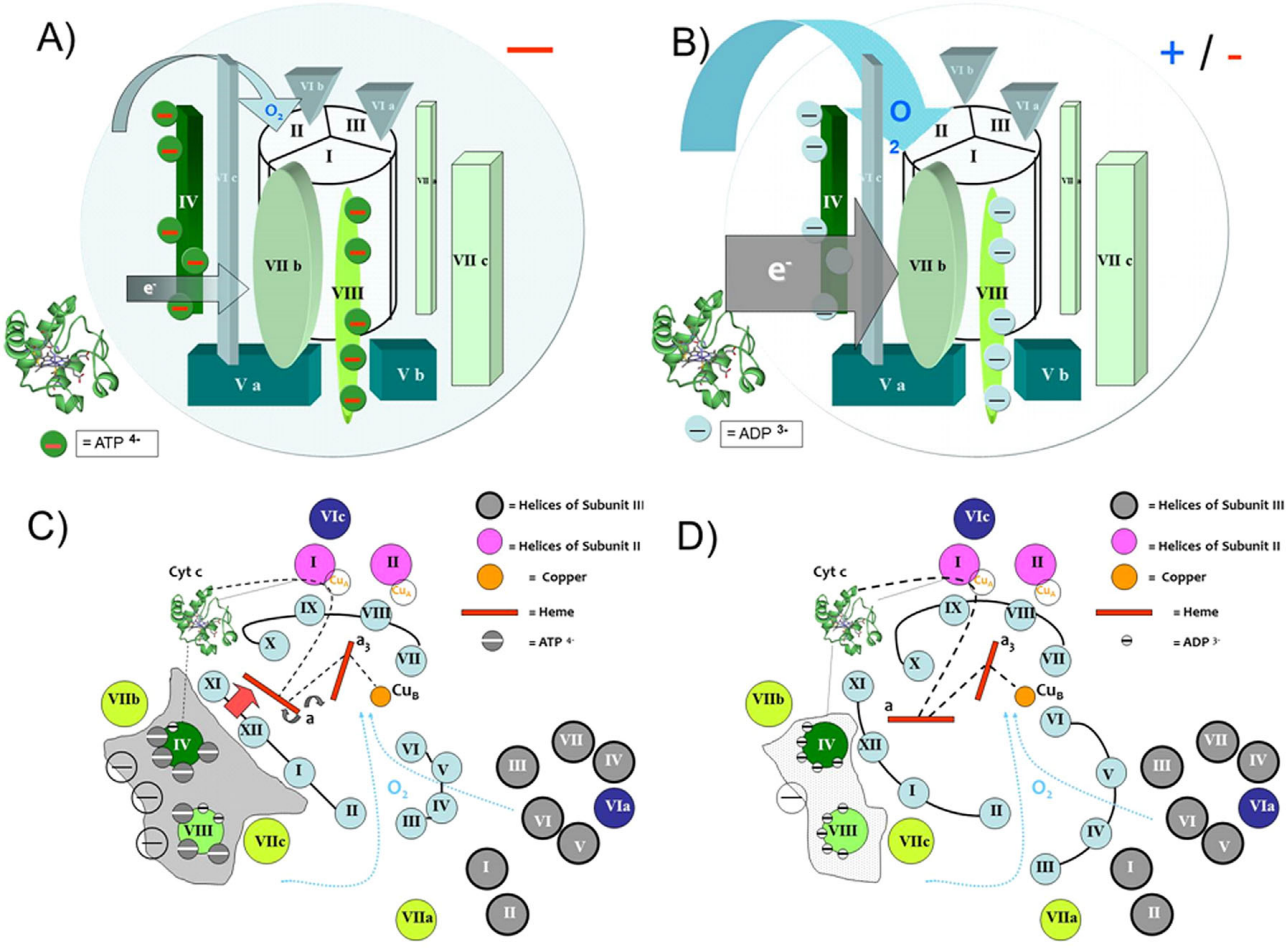
Schematic representation of the modified molecular structure of CytOx subunits, originally from Herrmann et al. 123. The mitochondrial‐encoded SU I, II and III have the central stage, whereas the nuclear‐ encoded SU surround the central column. The blue arrow represents the binding of oxygen to the transmembrane helices of SU I and II 124. Cytochrome c (molecule on the left, Hoffmeister K, Wikimedia commons) transfers electrons to CytOx (grey arrow ‘e^‐^’). It is proposed that during enzyme turnover the enzyme cycles between two conformers, one with a substrate binding site on subunit II, and the other along the interface of subunits II, IV and VIb. Structural analyses suggests that Glu112, Glu113, Glu114 and Asp125 of subunit IV, and Glu40, Glu54, Glu78, Asp35, Asp49, Asp73 and Asp74 of subunit VIb are residues that could possibly be involved 125. Cytochrome c binding affects the conformation of cytochrome a within CytOx 126. **A:** Proposed model representing the influence of ADP or ATP binding to SU IV and SU VIII on the enzymatic activity of CytOx. Ten binding sites for adenine nucleotides are known. At seven sites, ADP and ATP are exchanged 127. One binding site for ATP or ADP, located at the matrix‐oriented domain of the heart‐type subunit VIaH, increases the H+/e‐ stoichiometry of the enzyme in heart or skeletal muscle from 0.5 to 1.0 when bound ATP is exchanged by ADP. Two further binding sites for ATP or ADP are located at the cytosolic and the matrix domain of subunit IV. Although the additional binding site on SU VIa has been confirmed by Taanman et al. 128 (not shown), most binding sites were found on SU IV and VIII using radioactive ATP analogues, suggesting that these two nuclear‐coded polypeptides may play a regulatory role 129, 130. Especially, SU IV is essential for the assembly and respiratory function of the complete enzyme complex 131. Because of the negative charges associated with ATP (fourfold), and the dipole moment of cytochrome c 132, 133,the holoenzyme creates an electrostatic field (negative sign on the cycle) that finally regulates the internal electron‐transfer reactions by its electric field strength 134. This explains how CytOx acts like an ‘electro‐catalyst for oxygen reduction’ 135. Furthermore, Craig et al. 136, 137 and Lin et al. 138 found that ATP binding to cytochrome c diminishes electron flow in the mitochondrial respiratory pathway and respiration is shut down. **B:** In the case of the exchange of ADP to ATP on the seven nucleotide binding sites the electrostatic field becomes weaker because of less negative charge with ADP. Subsequently, electron transfer from cytochrome c to SU II becomes accelerated. **C:** Modified model for subunit order inside the CytOx molecule according Tsukihara et al. 139, 140 and shows again the proposed mechanism of ATP binding to SU IV and VIII. The subunits of CytOx in the molecule centre are shown with blue (SU I), pink (SU II) and dark grey cycles (SU III). Roman numbers represents the helices. Blue dotted lines mark the entry of Helix I/II/III to Oxygen pathway 1 and the entry of Helix IV/V to Oxygen pathway 2. The binding of ATP (small grey cycles with white minus signs) at seven positions to SU IV and VIII results in a higher negative charge for the molecular dipole. The more negative ‘cloud’ induces tilting and bending of the molecule, and the binding of cytochrome c (black dotted line) is influenced, resulting in alterations of the subunit positioning (here helices XI, XII, I and II) together with a reduction in the distance between haeme a and haeme a_3_. The influence of an electric potential field and the effect of ionic strength on the reaction rate of cytochrome c have been described by Koppenol et al. 141. **D:** The same molecular model features the situation after binding of ADP to all the binding sites of SU IV and VIII. A less negatively charged ‘cloud’ (left side) widens the distances between Helices XI, XII, I and II and finally induces a ‘more open’ angle between haeme a and haeme a_3_ for acceleration of electron transfer and increased Dioxygen turn over. However, the question of a pH‐dependent polarity change at the binuclear centre 142 remains unanswered, although the proton K‐pathway is known to become sufficiently flexible for internal water molecules to alternately occupy upper and lower parts of the oxygen pathways, which are associated with conserved Thr‐359 and Lys‐362 residues. Subsequent intramolecular ‘constrictions’ 143 could support the already known effect of dielectric relaxation of CytOx 144.

The basis of Kadenbach's theory is the proposal of a ‘second mechanism of respiratory control’ 50 that regulates the rate of respiration by the ATP/ADP ratio, extending the ‘first mechanism of respiratory control’, whereas excessively high values of Ψm ​​limit the respiration rate. Arnold and Kadenbach have shown that high intramitochondrial ATP/ADP ratios convert the hyperbolic kinetics of ascorbate‐dependent respiration of isolated CytOx to an inhibited allosteric kinetic status (Hill coefficient >1), which is independent of the Ψm and is based on the binding of ATP or ADP to the matrix domain of CytOx subunit IV 72. Ogbi et al. 73 observed that phosphorylation of CytOx subunit IV by protein kinase Cϵ produced an increase in the CytOx activity. They demonstrated that 4‐phorbol esters activate translocation of PKCϵ into mitochondria, which were immunoprecipitated with the CytOx subunit IV. The role of other subunits in this mechanism remains to be clarified. The subunits IV, VIa, VIb, VIIa and VIII are nuclear‐encoded subunits of CytOx and are expressed in 2 or 3 tissue‐ or development‐specific isoforms 74, 75, 76. The catalytic centre of the enzyme is located in three subunits that are encoded in the mitochondrial genome (haeme a and haeme a3/CuB in subunit I CuA in subunit II, see Fig. 2C and D). In addition to the catalytic subunits, the mammalian enzyme also contains 10 subunits hat are encoded by the nuclear genome, causing complex regulation of the enzymatic activity 52. Whether phosphorylation of subunits is involved, is still an open question. Western blot analysis of isolated CytOx (isolated under different conditions), using antibodies against phosphoserine and phosphothreonine, identified multiple phosphorylation sites on subunits I, II, III, IV, VIa, VIb, VIc, VIIa, VIIb and VIIc 9, 10. Allosteric inhibition of ATP is reversed by binding of thyroid hormone 3,5‐diiodo‐L‐thyronine, to CytOx subunit Va 77. Surprisingly, Lee et al. 78 observed that AMP‐dependent phosphorylated enzyme exhibited only allosteric ATP inhibition, which is abolished by Ca2 + ‐activated dephosphorylation 79. ATP‐dependent allosteric inhibition is postulated to maintain the mitochondrial membrane potential (Ψm) in vivo at low healthy values 9, 10; however, stressors can reduce the allosteric ATP‐inhibition of CytOx (e.g. due to calcium‐activated dephosphorylation or by 3,5‐diiodo‐L‐thyronine) thereby increasing the Ψm (beyond 140–200 mV) with ROS formation 37, 38. In numerous studies, a transient hyperpolarization of Ψm was described by those factors that trigger apoptosis in cancer cells, e.g. cytostatics, UV or laser light irradiation, staurosporine, oxidized LDL, overexpression of transglutaminase, p53, higher concentrations of free palmitate, high glucose concentration in neurons (diabetes), activation of the Fas receptor and hyperthyroidism. However, in none of the cases was the molecular cause for the hyperpolarization of Ψm described. The molecular‐physiological hypothesis of Kadenbach could explain these findings at a basic molecular level 54.

Recently, we observed that the ATP‐dependent inhibition of CytOx is also associated with ‘ischaemic preconditioning’ (IPC) 80, and thus again raises the question of the nature of the trigger. IPC has a cytoprotective benefit for the heart to help tolerate further ischaemic insults after subsequent potentially lethal ischaemia 18, 81. Surprisingly, a direct correlation with the phosphorylation patterns of the subunits was not found. Thus, ROS could act as a signal via phosphorylation of tyrosine kinases activating the nuclear transcription factor NFkB 82 and interaction with CytOx.

Preconditioning was observed after a brief ischaemia/reperfusion event, and may be generated experimentally by hypoxia, oxidative stress, heat shock or by activation of α1 receptors 83. IPC was reduced by mitochondrial *K*_ATP_ channels openers, i.e. dioxazid, pinacidil or nicorandil 40, 83, 84. Currently, the molecular basis of this effect is poorly understood 41, 85. Other studies relate to the opening of sarcoplasmic *K*_ATP_ channels 86, 87 and discuss the existence and involvement of mitochondrial K_ATP_ channels 88. In the case of a ‘late window’ after IPC, transcriptional activation of the expression of genes for heat shock proteins (HSPs) and oxidative stress‐degrading enzymes, such as superoxide dismutase (SOD), catalase, glutathione peroxidase and haeme oxygenase is assumed. The extent that IPC interferes with the ATP‐ dependent inhibition of CytOx is unknown.

### ATP‐dependent inhibition, allostery and phosphorylation sites of CytOx: Data remain controversial

Iksoo Lee was the first to demonstrate in her thesis an obvious correlation between the phosphorylation of cytochrome c oxidase by a cAMP‐dependent protein kinase A and ATP‐dependent allosteric enzyme inhibition 9, 10. By comparing consensus sequences, she suggested that this effect is triggered by phosphorylation of serine 441 in subunit IV. In contrast, Hüttemann and co‐workers 89 identified cAMP‐mediated inhibition of the enzyme, probably due to phosphorylation of tyrosine 304 on subunit I. An illustration from this original work; however, clearly shows that this phosphorylation is responsible for the rightward shift of the kinetics of the enzyme activity leading to the sigmoidal allosterically inhibited state but not to the theoretically expected transition of the enzyme kinetics to a hyperbolic state (Michaelis Menten kinetics 1st order). Another important observation was reported by Arnold and co‐workers 90. By a gradual reduction of oxygen, it was shown that transcription of CytOx subunit IV‐2 was induced specifically in astrocytes. Increased transcription of isoform IV‐2 caused an obvious switching off of the allosteric inhibition mechanism for CytOx in the presence of high concentrations of ATP. It was concluded that the presence of this isoform removes allosteric inhibition of the enzyme due to a reduced responsiveness to the allosteric regulator ‘ATP’. Therefore, an oxygen sensor function has been assigned to CytOx. Of course, different phosphorylation sites on the enzyme were examined in terms of their functional relevance 68, however, the identification and importance of the individual phosphorylation site responsible for the ATP‐dependent allosteric inhibition of CytOx remained in doubt.

Hüttemann and co‐workers 89 claimed that the allosteric ATP‐dependent inhibition of CytOx from bovine liver is related to the cAMP‐dependent phosphorylation of tyrosine 304 on the cytosolic side of the subunit I. Miyazaki et al. 91 demonstrated phosphorylation of subunit II of CytOx by a non‐receptor tyrosine kinase c‐Src in osteoblasts and found a positive correlation between CytOx activity and c‐Src kinase activity, although the amino acids that were phosphorylated remained obscure. A specific non‐receptor tyrosine phosphatase, SHP‐2, was detected by Salvi et al. 92 in mitochondria. Steenart and Shore 93 performed in vitro phosphorylation of CytOx subunit IV with [γ‐^32^P] ATP, but did not identify the phosphorylated amino acid. The signaling pathways leading to phosphorylation and modification of CytOx activity are still largely unknown. Hüttemann and co‐workers 89 have shown that the phosphorylation of Y304 in the CytOx subunit I is performed through G‐protein‐dependent receptors and that tyrosine phosphorylation of subunit IV is probably induced via the PI3 K (phosphatidylinositol 3 – kinase)/Akt (protein kinase B) pathway. Bijur and Jope 94 demonstrated phosphorylation of Akt after activation of PI3 K by IGF‐1 (insulin‐like growth factor) in cell cultures (SH‐SY5Y, HEK293) and further demonstrated that the phosphorylated Akt is translocated into mitochondria where it phosphorylates the β subunit of ATP synthase, glycogen synthase kinase‐3β and other unknown proteins. The Manfredi group identified another important pathway for cAMP action concerning regulation of oxidative phosphorylation 67. They proposed an intramitochondrial CO_2_‐HCO_3_ **^−^‐**sAC‐cAMP‐PKA regulatory pathway for oxidative phosphorylation. The latest findings of Hess et al. indicate a ‘CO_2_‐HCO_3_^−^‐sAC‐cAMP‐ signalosome’ that is responsible for PKA activation and phosphorylation of subunit Va at positions T65 and S 43 of CytOx in *Saccharomyces cerevisiae* under normoxic conditions 95. These phosphorylations modulate the allosteric regulation of CytOx by ATP and the authors showed that the normoxic subunit Va is a homologue of human subunit IV‐1 (isoform), but the same experiments in human systems have yet to be performed. Acin‐Perez et al. 96 demonstrated that residue S56 in mammalian CytOx subunit IV‐1 is coupled with the prevention of allosteric inhibition of CytOx by ATP. In addition to discussions concerning phosphorylated residues of CytOx 97 these data demonstrate the allosteric inhibition of CytOx by ATP and confirms part of Kadenbach's theory. We have already shown a relationship between the ATP‐dependent inhibition of CytOx and decreased ROS production 98. Finally, the question remains whether all the ATP‐dependent inhibitory effect of CytOx is always associated with allostery and for additional factors causing allostery.

Yaniv et al. 99 found that cAMP/PKA signaling is dependent on Calcium regulation. Effects on mitochondrial metabolism are due to the activation of soluble mitochondrial Adenylyl Cyclase by bicarbonate and calcium 100. However, conflicting data were also published by the Balaban group. They observed a stimulation of oxidative phosphorylation by calcium without an influence by cAMP and PKA activity 101. The pH dependency of bicarbonate‐regulated soluble Adenylyl Cyclase 102 remains to be clarified in the context of the inhibitory effect of ATP on CytOx. Finally, Acin‐Perez et al. 103 described a Phosphodiesterase 2 A that is localized in mitochondria and is involved in the regulation of respiration. This type of PDE2A is located in the matrix. Concerning different signaling chains for protein phosphorylations 104 and multiple phosphorylation sites of CytOx 105, 106, and the ‘so far known’ compartmentation of cyclic nucleotide signaling 107 on the other hand, we have to address the question whether all the different cAC actions 108 are maintained by a network of different PDE's in the mitochondria or in the intramembranous space 109.

### Phosphodiesterase inhibitors as true regulators?

**Figure 3:**
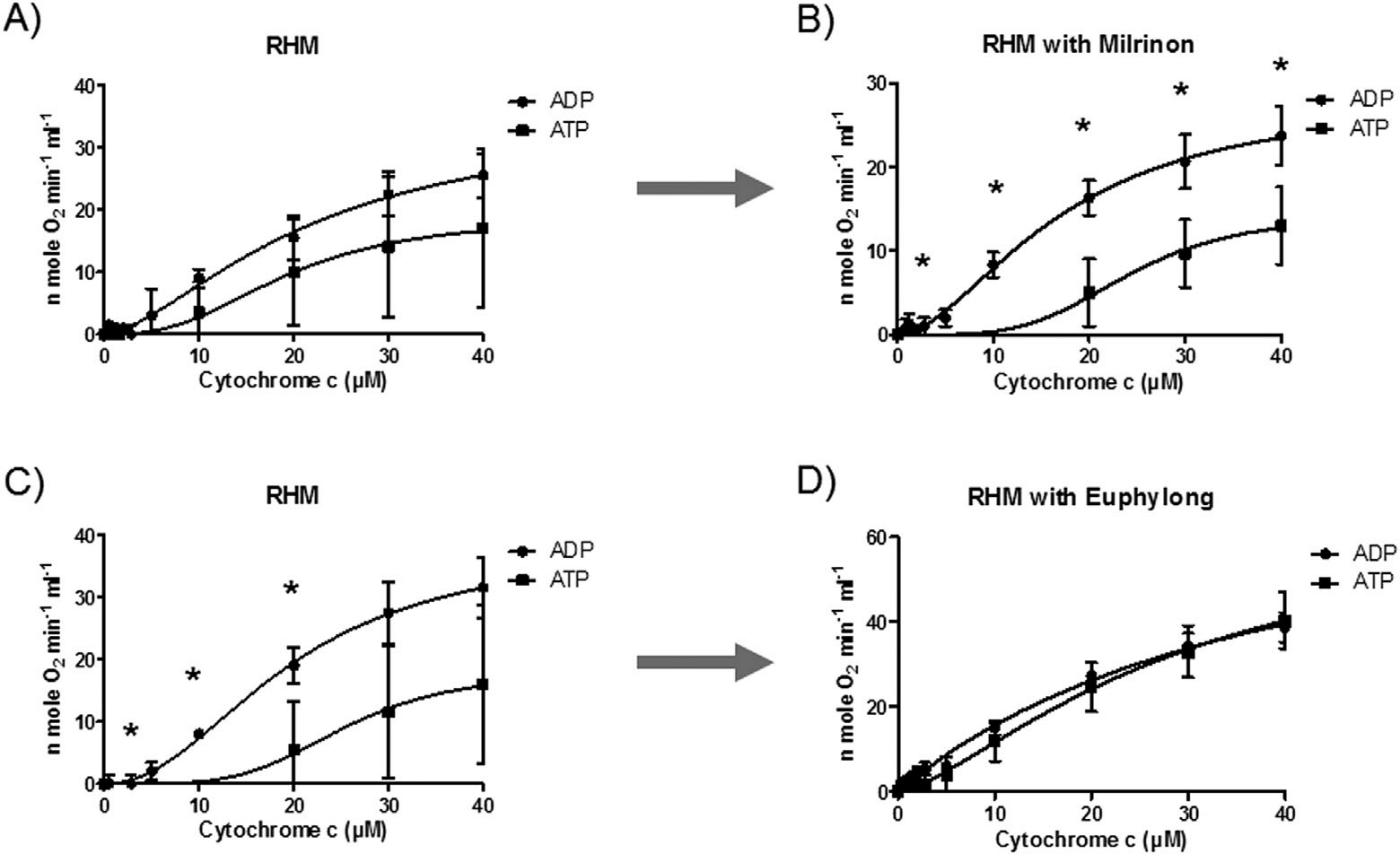
Freshly isolated rat heart mitochondria were used for the kinetic measurements of CytOx activity without (**A**) and with 1 μg/mL Milrinone (**B**), and without (**C**) and with 1 μg/mL Euphylong (**D**). Each phosphodiesterase inhibitor was added directly to the sucrose‐containing buffer. Oxygen consumption as a function of CytOx activity was measured in the presence of either 5 mM ADP or 5 mM ATP + 10 mM Phosphoenolpyruvate and 160 U/mL pyruvate kinase at increasing concentrations of cytochrome c (0–40 μM) using 18 mM ascorbate as a substrate. Mitochondrial stock protein concentrations were determined by the BCA method and were 26.6 ± 1.30 mg/ml (*n* = 3). Concentrations of mitochondria between Figure A and C were 5 to 1. *Wilcoxon‐Mann‐Whitney‐rank sum test *p* < 0.05.

Regarding the data from the Manfredi group, Lee and co‐workers studied signaling pathways targeting mitochondria and examined phosphorylation of CytOx subunits by the cAMP‐dependent pathway. Using phospho‐antibodies against phospho‐tyrosine, they detected phosphorylated cow liver CytOx subunit I in the presence of theophylline, a phosphodiesterase inhibitor (PDE inhibitor) that induces high levels of cAMP. This type of phosphorylation of Tyr304 in CytOx decreased V(max) and increased K(m) for cytochrome c. It shifted the reaction kinetics from hyperbolic to sigmoidal as CytOx is fully or strongly inhibited up to 10 μM concentrations of cytochrome c 89. Phosphodiesterase inhibitors (PDE) are known from their use in therapy of cardiovascular diseases, e.g. treatment of cardiac insufficiency. A wide spectrum of pharmaceuticals display their actions directly or indirectly on the status of mitochondrial bioenergetics. Surprisingly, our research group observed that the drugs Milrinone (PDE III inhibitor; 2‐methyl‐6‐oxo‐1,6‐dihydro‐3,4′‐bipyridine‐5‐carbonitrile) and Euphylong (Theophylline; 1,3‐Dimethylxanthin) had an opposite effect on CytOx kinetics (Fig. 3A–D). Allosteric inhibition was intensified by Milrinone, whereas Theophylline reversed this inhibition completely. These beneficial effects of Theophylline on ischaemic tissues act in a dose‐dependent manner 110. Milrinone treatment in cases of severe cardiac failure appears in a new spotlight 111 because myocardial dysfunction after ischaemia /reperfusion 35, 112 could be prevented by administration of Milrinone 113. PDE networks appear confusing. Inhibitors of PDE, which cause increased concentrations of cyclic nucleotides, are expressed in multiple tissue‐specific isoforms 114. Until recently, 21 human PDE genes had been identified with 11 families and more than 60 known isoforms and more than 20 crystal structures had been identified. PDE's increase cellular cAMP and/or cGMP levels, and thus are involved in the regulation of numerous cAMP‐and cGMP‐dependent signaling pathways, such as metabolism and gene expression. PDE3 binds cAMP with a higher affinity than with cGMP. However, PDE4, PDE7 and PDE8 react only with cAMP, whereas PDE5, PDE6 and PDE9 react with cGMP; the classical PDE inhibitors theophylline (1,3‐dimethylxanthin) and IBMX (3‐isobutyl‐1‐methylxanthine) seem relatively nonspecific. As a result of these data, a cellular regulatory network with mitochondrial competence can be proposed; however, PDE inhibitors with high affinities for isoenzymes are still desired 115. The actions of dipyridamole (an inhibitor of PDE6, PDE7, PDE8, PDE10 and PDE11), milrinone (both PDE3 inhibitors) and sildenafil (inhibitor of PDE1, PDE5 and PDE6) are less selective.

### Conclusions and outlook

The inhibition of CytOx by ATP presents a ‘second mechanism of respiratory control’ 50, which regulates the respiration rate by the ATP/ADP ratio, supporting the ‘first mechanism of respiratory control’, whereas the respiratory rate is limited at high ΔΨm values. When ATP is bound to CytOx, the enzyme is inhibited, and there is subsequently low formation of ROS, although the mitochondrial membrane potential is increased. The initial data from the Kadenbach group were confirmed, whereas the *sigmoidal* kinetics of the enzyme at high intramitochondrial ATP/ADP ratios with ADP bound to the CytOx subunit IV and replaced by ATP remains to be clarified 72. Allosteric inhibition by ATP indicates a blockage of CytOx enzymatic activity at high concentrations of ATP, which is likely removed by dephosphorylation of CytOx as a result of activation of a calcium‐dependent protein phosphatase 79. However, the different phosphorylation steps of CytOx subunits are not completely understood. The allosteric inhibition of CytOx by ATP represents a control circuit at low ΔΨm values, which maintains the mitochondrial membrane potential within a physiological range. It has been confirmed that ATP binding to CytOx diminishes electron flow in the ETC 116. In experiments with 8‐Azido‐ATP‐ modified CytOx and with Cytochrome c, modulation of electron transfer from Cytochrome c to CytOx by interacting with the enzyme and allosterically altering the docking was confirmed. However, if binding of ATP affects primary Cytochrome c or CytOx or both, reduced electron transfer remains open 117, 118, 119. However, in fact, the docking scenario of Cytochrome c to CytOx under the influence of ATP is changed. Whether the ATP‐cytochrome c adducts have a different binding site or a different docking conformation remains to be demonstrated. It is worth noting that the influence of Cardiolipin is not negligible. Tuominen et al. 120 found that ATP induction of conformation alterations was dependent on binding of lipid to Cytochrome c via an Arg^91^‐containing binding site. Cytochrome c bound to Cardiolipin and ATP has a high level of Peroxidase activity that favours protein structures with an open haeme pocket 121 (see Fig. 2C and D). Therefore, we suggest both an electron scavenging effect and a modification of subunit I containing the two haeme centres on CytOx by ATP binding (see Fig. 2 A and B). Haeme a acts as an ‘opened or closed baseball glove’ catching as an electron input device. Haeme a_3_ acts as part of the binuclear centre and site of oxygen reduction (Fig. 2A–D). Kadenbach's theory postulates that stress removes ATP‐dependent inhibition of CytOx 54, resulting in an increase in the ΔΨm and excessive formation of ROS 37. However, the relationship between the ATP‐dependent inhibition of CytOx enzyme activity, an increase of the ΔΨm and formation of ROS as a mitochondrial regulator is not yet known. We have observed that the rate of electron transmission on CytOx determines the inhibitory effect and the assumed subsequent production of ROS, which are generally considered a major cause of tissue damage 26, 27. Thus, degenerative diseases and ageing could be better understood as an elementary mitochondrial process. Although debatable, phosphodiesterase inhibitors appear as key regulatory factors that influence the respiratory activity of CytOx. It is likely that analysis of PDE action can provide a framework for further studies because a variety of chemical compounds may affect oxidative phosphorylation to a much larger extent 122.

The authors declare no conflict of interests regarding the publication of this paper.
